# Demographic and clinical variables in the dengue epidemic in Punjab, Pakistan

**DOI:** 10.12669/pjms.39.6.7383

**Published:** 2023

**Authors:** Saira Mushtaq, Malik Ihsan Ullah Khan, Muhammad Tahir Khan, Aamir Husain

**Affiliations:** 1 Saira Mushtaq, M.Phil. Biochemistry Institute of Molecular Biology and Biotechnology, The University of Lahore KM Defence Road, Lahore, Pakistan; 2 Malik Ihsan Ullah Khan, Ph.D. Biology Institute of Molecular Biology and Biotechnology, The University of Lahore KM Defence Road, Lahore, Pakistan; 3 Muhammad Tahir Khan, Ph.D. Bioinformatics Institute of Molecular Biology and Biotechnology Research Scientist at the Zhongjing Research and, Industrialization Institute of Chinese Medicine, Zhongguancun Scientific Park, Meixi, Nanyang, Henan, 473006, PR China; 4 Aamir Husain, FCPS Medicine Allied Hospital, Faisalabad, Pakistan

**Keywords:** Dengue fever, Pakistan, Demographic features, Clinical symptoms, Aspartate Transaminase, Alanine Transaminase, Alkaline Phosphatase

## Abstract

**Objectives:**

To identify the latest trends in the clinical picture and severity of the disease, which will help better understand and manage dengue.

**Methodology:**

It was a cross-sectional, hospital-based study performed in the tertiary care hospitals of Punjab from August 21 to December 2022, in which serologically and polymerase chain reaction (PCR) confirmed patients with dengue infection, were enrolled. Demographic and clinical variables were recorded on a pre-tested Performa, processed and presented in frequency and percentages, and graphs were generated. Mean and standard deviation was used to present continuous variables.

**Results:**

Out of a total of 580 patients, 472 were diagnosed with Dengue Fever (DF) and 108 with Dengue Hemorrhagic Fever (DHF). About 79.31% of the patients were male and 20.69% were females. The mean age of patients was 32.5±9 years. Among the clinical features the percentage of high-grade fever, body aches, and vomiting were the highest. The liver function profile showed that serum bilirubin, Serum aspartate transaminase (AST), serum alanine transaminase (ALT,) and alkaline phosphatase (ALP) levels were markedly raised.

**Conclusion:**

This study showed that with time the trends in the presentation of dengue are slowly shifting, which will help us better manage the disease burden in the future.

## INTRODUCTION

Dengue is a critical febrile disease that manifests itself after three to ten days of a dengue virus (DENV) infected mosquito bite.^1^ An estimated 390 million people get infected with the dengue virus yearly worldwide, out of which 96 million show clinical manifestations.^2^ Pakistan has been experiencing an epidemic of dengue fever every year with a peak in intensity after the rainy season. Majority of the patients are in Khyber Pukhtunkhawa, Punjab and Sindh provinces of Pakistan.^3^ The median age of DF patients in Pakistan falls between 32 to 24 years of age and they are predominantly of female gender.^4^,^5^

Patients present with high fever, rashes, headache, body aches especially in joints, nausea, and vomiting.^6^ In addition, thrombocytopenia,^7^ leukopenia, and deranged liver function tests were also positive in Pakistan.^8^ This study was designed to observe and report the demographical parameters like age and gender involvement and clinical and diagnostic presentation of dengue disease in the latest epidemic of 2021. This will enable us to do a comparison of current and previous epidemics thus giving us a better understanding of current trends in the disease and how to effectively manage them.

## METHODS

It was a cross-sectional, hospital-based study performed in the tertiary care hospitals of Lahore, Faisalabad, Multan and Sargodha, Punjab from August 21 to November 2022. A total of 580 Confirmed patients of dengue infection with symptoms like fever, retro-orbital pain, headache, body pains, haemorrhagic manifestations with low platelet count (<100,000/UL) and low white blood cell count (<4000/UL) were enrolled in the study. Detailed clinical history, findings of clinical examination, and laboratory and other diagnostic test results were recorded on a pre-tested Performa. Patients were labelled with DHF if there was fever, haemorrhagic manifestations, low platelet count, an elevated haematocrit of more than or equal to 20%, pleural effusion, or ascites.^9^

### Ethical Approval:

Ethical clearance for the current study was taken from the Institution of Molecular Biology and Biotechnology Bioethical, Biosafety and Biosecurity Committee, The University of Lahore (Letter No: Ref_CRiMM/22/Research/143).

They were confirmed of dengue infection by either a positive polymerase chain reaction (PCR) test for DENV or non-structural protein-1 (NS1) antigen positive or show positive immunoglobulin M (IgM) antibodies for DENV. They were of both genders and were of more than 13 years in age. Patients suffering from comorbidities like hepatitis, chronic liver disease, typhoid fever, or malaria were excluded from the study. Patients with dengue shock syndrome (DSS) were also excluded. Written consent was taken from these patients.

The data collected from multiple sources was combined in an excel file, with separate sheets for each city. A small python script using the ‘Pandas’ library was applied to process and analyse the data. The code reads data from the excel file and calculated required statistics. Variables like age, gender, clinical signs and symptoms, laboratory, x-ray, and ultrasound findings were processed and the counts for each demographic were stored on a new excel file. SPSS version 20.0 was used for analysis of the data obtained. The data of categorical variables was presented in frequency and percentages, and graphs were generated for them. Mean and standard deviation was used to present some continuous variables.

## RESULTS

Among 580 patients enrolled in the study, 472 were diagnosed as DF and 108 as DHF. About 79.31% of the patients were male and 20.69 % were females. The mean age of patients was 32.5±9 years. The age group 20 to 30 years showed the highest frequency and percentages of dengue virus infection and was followed by the age group 30-40 years, 40 to 50 years, and 10-20 years. Small percentages of individuals with age 50-60 years were also observed (Table-I). Among the sign and symptoms frequency and percentage of high-grade fever are very high (97%), followed by body aches (74%), vomiting (40%), fatigue (34%), abdominal pain (31.5%), bleeding (21%) and pleural effusion (15%). Besides the low frequency and percentages of symptoms diarrhoea, ascites, cough, oral bleeding, bleeding from the nose, vagina, vomit, stool, and urine were also observed.

**Table-I:** Frequency and percentages of different age groups n=584.

Age	Frequency	Percentage (%)
< 10 years	0	0.00
10 - 20 years	78	13.45
21 - 30 years	250	43.10
31 - 40 years	108	18.26
41 - 50 years	82	14.14
51 - 60 years	40	6.90
60 - 70 years	22	3.79

*Number of patients, n=580.

The haematological laboratory findings of all dengue patients showed that the most common abnormality was low platelet count (96%) followed by low total leukocyte count (TLC) (60%). Liver function profile analysis is given in Table-II and III. It showed that serum bilirubin level was raised in only 4% of patients. The mean value of it was raised more in DF patients (1.24) than in DHF patients (0.93). Serum aspartate transaminase (AST) levels (91%) were increased more in patients than serum alanine transaminase (ALT) levels (73%). DF patients had AST and ALT levels of 184.4 and 119.6, whereas DHF patients had mean AST and ALT levels of 578.75 and 164.4. This showed that both parameters were more raised in DHF patients than in DF patients, showing more liver damage in DHF patients. Alkaline phosphatase (ALP) levels were high in 51% of patients, being more markedly raised in DHF patients (mean of 198.31) than in DF patients (mean of 142).

**Table-II:** Liver function profile of dengue virus-infected patients.

Lab test	Normal	High	Low
	Count (%)	Count (%)	Count (%)
Serum Bilirubin Total mg/dL	402 96	18 4	0 0
AST U/L	22 9	216 91	0 0
ALT U/L	132 27	362 73	0 0
ALP U/L	120 49	126 51	0 0

*(U/L: units per litre), Number of patients, n=580.

**Table-III:** Comparison of Liver parameters in DF and DHF.

		Count	Mean	Standard Deviation	Min	Median	Max
DHF	Serum Bilirubin mg/dL	66	0.930303	0.64355	0.44	0.7	2.7
	AST U/L	40	578.75	1116s.221873	98	264	4887
	ALT U/L	90	164.4	229.202271	25	99	1434
	ALP U/L	38	198.315789	155.692022	60	152	524
DF	Serum Bilirubin mg/dL	354	1.241412	5.337047	0.19	0.6	51
	AST U/L	198	184.444444	148.553924	14	145	714
	ALT U/L	404	119.613861	107.604041	14	91.5	624
	ALP U/L	208	142.076923	81.20121	25	132.5	512

*Total number of patients, n=580, DF n=472, DHF n=108.

## DISCUSSION

Dengue has become a major public health issue internationally in recent years. It has four spectra of disease ranging from asymptomatic infection to DF, DHF, and DSS. In this study, a total of 580 patients fulfilling inclusion criteria were enrolled. Out of those, 472 were diagnosed as DF and 108 as DHF. An important risk factor for severe disease after infection with dengue virus is age. In our study, among the patients enrolled the age group 20 to 30 years showed the highest percentage of dengue virus infection. This is supported by another study in which the median age of dengue fever was 36 years.^10^ Similar findings were presented in a study done by Mehmood et al.^11^ in which the mean age of dengue fever patients was 34.4 (SD 14.4) years. Iqtadar et al conferred in their research that the mean age of the patients was 33.2±9 years, which supports the findings of this study.^12^ This shows that the age range getting affected by dengue is progressively decreasing and our study confirmed the same pattern.

According to our study dengue fever is more prevalent (79.31%) in males as compared to females (20.69%). This is in consistence with another study that found males suffering from the disease more than females.^13^ Male predominance may be due to many reasons like outdoor activities of males are more than females. Dressings of males especially in summer may be a factor in viral infection.^14^ Our study observed that among the sign and symptoms of dengue infection, the percentage of fever is very high (97%), followed by body aches, vomiting, fatigue, and abdominal pain. Besides these low frequency and percentages of diarrhea, cough, bleeding from nose, mouth, vagina, vomit, stool, and urine were also observed. This is positively reinforced by another study which established that the most known signs and symptoms were fever, myalgia, headache, and retro-orbital pain.^11^ Another study also demonstrated that signs and symptoms of dengue fever were high-grade fever, vomiting, abdominal pain, ascites, pleural effusion, GIT bleeding, gum bleeding, and skin bleeding.^15^

A comparative study conducted in Bangladesh showed an increasing incidence of abdominal symptoms like abdominal pain anorexia, and diarrhea whereas a decreasing tendency was noticed in terms of hemorrhagic manifestations including melena, conjunctival hemorrhage, hemorrhagic sclera, or skin rash.^16^ This was in affirmation of our findings. We observed in our study that very few patients (1.3%) develop rashes. However, literature cited in 1989 showed that 50 to 82% of patients reported skin rash^17^, while a study published in 2018 showed that out of 331 patients 55 developed rash.^18^ Dengue patients without skin rash develop complications and poor outcomes.^19^ This may lead us to conclude that as the incidence of skin rash decreases the severity of dengue infection increases.

In our study the most common abnormality among hematological laboratory findings was low platelet count (96%) followed by low TLC (60%). These findings are consistent with other studies which showed decreased platelet count in dengue fever patients.^20^ Low platelet count was seen in 85% of patients in an analogous study done in Karachi.^21^ The cause of thrombocytopenia in DF may be due to bone marrow depression, the DENV causing accelerated destruction of platelets, and the presence of antibodies directed against platelets.^22^

Dengue is found to have a predominant effect on the liver resulting in deranged liver enzymes. The results of our study showed that serum bilirubin level was raised in only 4% of patients. The mean value of it was raised more in DF patients than in DHF patients. In a study carried out in Malaysia similar pattern was observed. Hyperbilirubinemia was noted in 12% of DHF and 8% of DF patients. The mean (range) serum bilirubin was higher in DHF as compared to DF.^23^ Another study also showed serum bilirubin levels to be only marginally elevated in acute dengue patients.^24^ In our study, both serum AST and serum ALT levels were markedly increased in dengue patients. DF patients had an AST and ALT levels of 184.4 and 119.6, whereas DHF patients had a mean AST and ALT levels of 578.75 and 164.4. Both parameters were more raised in DHF patients than in DF patients, showing more liver damage in DHF patients.

A study conducted by Lee et al. showed that there was a relevance between raised liver enzymes and liver damage and serum AST and ALT both were raised more in DHF patients than in DF patients.^25^ Iqtadar et al. also concluded in their study that in dengue patients’ liver involvement is not limited to raised AST and ALT only, serum bilirubin levels and ALP are also raised considerably.^12^ In another few studies, the average raised levels of AST were in the range from 93.3 IU/L to 174 IU/L while ALT levels were from 86 IU/L to 88.5 IU/L.^26^ Ahmed in their study on the correlation of liver enzymes and severity of dengue infection found that AST levels were twice as elevated as ALT levels.^27^

According to the WHO report 2022, in Pakistan a total of 25, 932 confirmed cases and 62 deaths have been reported, among which 74% occurred in the month of September alone.^3^ The current increase may be due to the flooding in June, 2022. With the current flood a high-risk health impacts are expected from DENV fever. Punjab is the largest province of Pakistan by population based and second largest by area, located in central-eastern region of the country.^28^ For this purpose a detailed study to quantify all the and clinical variables was need of time to find out the current disease pattern. Our study has provided a recent and updated record of all important dengue fever parameters, which will help physicians to better handle the current disease.

### Limitations:

It includes lack of data due to poor history taking by physicians, and incomplete laboratory testing done due to the non-availability of facilities and funds in government-run tertiary care hospitals.

## CONCLUSION

The age range affected the most by this disease is decreasing and most patients belong to the ages between 20 to 30 years and the gender involvement is predominantly male. Skin rash and retro-orbital pain presented in very few patients. The severity of liver disfunction is increasing over time.

### Abbreviations:

**DENV**: Dengue virus

**DF:** Dengue fever

**DHF:** Dengue hemorrhagic fever

**AST:** Aspartate transaminase

**ALT:** Alanine transaminase

**ALP:** Alkaline Phosphatase

**TLC:** Total Leukocyte Count

**GIT:** Gastrointestinal tract

**DSS:** Dengue shock syndrome

### Authors’ Contribution:

**SM:** Preparation of manuscript, data collection, methodology, result compilation and responsible for the integrity and accuracy of the study.

**MIUK:** Supervision, provision of resources during data collection

**MTK:** Supervision, the main conceptualization of project, defining aims and objectives.

**AH:** Support in providing resources, supervision, questionnaire preparation.
